# Generation of induced pluripotent stem cells from the Asian bats

**DOI:** 10.1080/23144599.2024.2384835

**Published:** 2024-08-12

**Authors:** Younsu Lee, Okjae Koo, Islam M. Saadeldin

**Affiliations:** a Division of R&D, RedGene Inc, Seoul, Republic of Korea; b College of Veterinary Medicine, Kyungpook National University, Daegu, Republic of Korea; c nSAGE Inc., Incheon, Republic of Korea; d Comparative Medicine Department, King Faisal Specialist Hospital and Research Centre, Riyadh, Saudi Arabia

**Keywords:** Native bats, induced pluripotent stem cells, reprogramming, chimera

## Abstract

Preservation of native Korean bats is crucial for maintaining ecological balance, as they play a vital role in insect control, pollination, and seed dispersal within their ecosystems. The present study details the establishment of bat induced pluripotent stem cells (BatiPSCs) from two Asian and Korean bats (*Hypsugo alaschanicus* and *Pipistrellus abramus*) using the Sendai Reprogramming Kit. Colonies of BatiPSCs, exhibiting distinctive features, were manually selected and expanded following successful transfection. Characterization of BatiPSCs revealed the expression of pluripotency markers, such as Octamer-binding transcription factor 4 (Oct4), SRY (sex-determining region Y)-box 2 and Nanog, with notably increased Oct4 levels and reduced Myc proto-oncogene expression compared with those noted in other induced pluripotent stem cell sources. BatiPSCs displayed positive staining for alkaline phosphatase and demonstrated the ability to form embryoid bodies, while also inducing teratomas in non-immune nude mice. Additionally, green fluorescent protein (GFP)-expressing BatiPSCs were generated and used for chimeric mouse production, with slight GFP signals detected in the neck region of the resulting mouse foetuses. These findings demonstrate the successful generation and characterization of BatiPSCs, emphasizing their potential applications in chimeric animal models, and the protection of endangered bat species.

## 1. Introduction

Induced pluripotent stem cells (iPSCs) play a promising role in the conservation of wildlife species particularly bats by providing a versatile tool for genetic research, disease modelling, and potential restoration efforts [1–3]. The conservation of these bats also supports broader ecosystem functions and resilience, ensuring the sustainability of natural habitats and the services they provide to humans and other wildlife [4].

These iPSCs pose the unique ability to differentiate into any cell type in the living body, making them an invaluable resource for disease modelling, drug discovery and personalized cell-based therapies [5–7].

iPSCs are generated through a process called cellular reprogramming, where somatic cells are reprogrammed back into a pluripotent state. The reprogramming process involves the introduction of specific transcription factors known as Yamanaka factors. These factors are the following: Octamer-binding transcription factor (Oct) 4, SRY (sex-determining region Y)-box 2 (Sox2), Kruppel-like factor 4 (Klf4) and Myc proto-oncogene (c-Myc), which are present in somatic cells and their function is to reset their gene expression patterns and induce pluripotency [6,7]. The collective action of these factors leads to the activation of endogenous pluripotency genes, such as Nanog and Rex1, and the silencing of lineage-specific genes, ultimately reprogramming the somatic cells into iPSCs.

Following the initial reprogramming phase, the generated iPSCs undergo a series of culture and expansion steps to establish stable and self-renewing cell lines. These cells exhibit key characteristics of embryonic stem cells, including the ability to self-renew indefinitely and differentiate into cells of all three germ layers (endoderm, mesoderm and ectoderm). iPSCs can be maintained in culture for long periods, providing an abundant and accessible source of pluripotent cells for various applications [5,8].

The retroviruses or lentiviruses are commonly used as vectors to deliver these factors into the target cells. However, in recent years, advancements have been made in reprogramming techniques to enhance efficiency and reduce potential risks associated with the use of viral vectors. Non-integrating methods, such as episomal vectors, mRNA transfection and protein delivery systems, have been developed to avoid permanent genetic modifications and improve the safety profile of iPSC generation [9,10].

The global impact of the coronavirus disease-19 (COVID‐19) outbreak, which began in late 2019, has extended over a span of more than 2 years, resulting in significant repercussions on both global quality of life and economical status. The focus has intensified on unravelling the origins and transmission of the severe acute respiratory syndrome coronavirus 2 (SARS‐CoV‐2). Currently, the discourse centres on the following two conflicting theories: The possibility of laboratory spillover events and human interaction with zoonotic diseases originating from the bats [11]. To achieve the 3 R (replacement, reduction, and refinement) principle, iPSc can be an alternative to animal models for studying disease progression, evaluating therapeutics and vaccines and guiding public health interventions [12–14].

An earlier report indicated the generation of bat iPSCs [15] and a recent report demonstrated their ability to form chimeric embryos [2] and their use in coronavirus disease-19 (COVID-19) research [1]. The current work provides a comprehensive overview of the generation of BatiPSCs, highlighting their immense potential in preserving the native Korean bats and for further clinical practice.

## 2. Materials and methods

The present study was carried out with a permission from Yeongju City, Gyeongsangbuk-do, Republic of Korea to capture and collect wild animals (permit number: 509000058201500001). Animal treatment and maintenance were reviewed and approved by the institutional animal care and use committee (IACUC) of Institute for Basic Science (IBS), Yuseong-gu, Daejeon, Republic of Korea.

### 2.1. Chemicals and reagents

Chemicals and reagents were obtained from Sigma-Aldrich (Merck KGaA) unless otherwise specified.

### 2.2. Establishment of primary bat fetal fibroblast (PBFF)

Foetuses were detected through manual abdominal palpation [16]. Euthanasia of bats was performed using a gradual CO_2_ charging method (70% chamber volume/min), according to [17,18] and the Michigan Rabies Working Group (RWG) [19]. After waiting until the bat in the chamber completely lost breathing and consciousness, it was euthanized after waiting for another 3–5 minutes. The bat was then taken out and confirmed dead with no heartbeat through palpation. PBFFs were isolated from the foetal stage of two native Korean bats [*Hypsugo alaschanicus* (*n* = 4) and *Pipistrellus abramus* (*n* = 4)] with an average gestation period is 7.5 weeks [20,21]. The foetuses’ heads and internal organs were removed and the remaining tissues were washed with phosphate-buffered saline (PBS). Subsequently, the tissues were minced into small pieces using scissors and digested with a 0.25% trypsin/1 mM EDTA solution in a 37°C water bath for 15 min. Following trypsinization, equal volumes of mouse embryonic fibroblast (MEF) medium (DMEM with 10% FBS) were added and pipetted to dissociate the cells [1]. The supernatant containing dissociated cells was collected and filtered through a polyvinylidene fluoride filter with a pore size of 0.45 μm (Millipore, Sigma). The cells were subsequently collected by centrifugation at 1,000 × g for 3 min and resuspended in MEF medium. The cells were passaged two or three times to establish a morphologically homogeneous culture. Subsequently, the cells were either frozen for preservation or expanded for further experimental studies.

### 2.3. Generation of bat iPSCs

PBFF cells were reprogrammed using the integration-free CytoTune®-iPS 2.0 Sendai Reprogramming Kit (Thermo Fisher Scientific, Inc.). This kit includes Sendai viral particles carrying the Yamanaka factors (human Oct3/4, Sox2, Klf4, c-Myc). To initiate the reprogramming process, 3 × 10^5^ BEFs were seeded onto a gelatin-coated well of 6-well plate 24 h prior to viral transduction. The cells were transduced in accordance with the manufacturer’s protocol. Nine days post-transduction, small colonies were manually transferred with stretched glass Pasteur pipettes onto mouse embryonic fibroblast (MEF) feeder cells (Cat# SCRC-1008, ATCC, Manassas, VA, USA) and cultured using Stempan E14 GMEM (Product # P08–50600, PAN-Biotech GmbH, Aidenbach. Germany) or TeSR™-E8™medium (Catalog #05990, Stem Cell Technologies, Vancouver, BC, Canada). The colonies were subsequently dissociated using 0.1% trypsin/1 mM EDTA solution and passaged onto new 6-well plates. Upon reaching approximately 60–70% confluency, individual cells were expanded onto 10 cm dishes.

### 2.4. Characterization of bat iPSCs through immunofluorescence

Immunofluorescence analysis was conducted following a standard protocol. Briefly, the cells were fixed in 4% paraformaldehyde (Merck KGaA), washed three times with PBS and subsequently permeabilized with 0.3% Triton X-100 (Merck KGaA) and blocked with 5% normal foetal calf serum for 1 h at room temperature. The cells were subsequently incubated with primary mouse antibodies against Oct4 (P0082-200UL, Sigma-Aldrich), and primary rabbit antibodies against Nanog (RCAB004P-F, ReproCELL, Beltsville, MD, USA) at 4°C overnight. Primary antibodies were diluted 1:100 in 5% normal foetal calf serum. Following washing three times with PBS, the cells were incubated with secondary antibodies (Alexa CytoTune® 594 goat anti-mouse IgG (Cat # A-11005) and Alexa TeSR™ 546 goat anti-rabbit IgG (Cat # A-11035), Thermo Fisher Scientific, Inc.) for 2 h at room temperature. Secondary antibodies were diluted 1:200 in PBS. The nuclei were counterstained with 4,6-diamidino-2-phenylindole-DAPI (Cat# 28718-90-3, Sigma-Aldrich). To exclude autofluorescence and non-specific binding of secondary antibodies, controls without primary antibodies were conducted, maintaining the same incubation durations.

### 2.5. Characterization of bat iPSCs through alkaline phosphatase (AP) reaction and embryoid body formation

For the detection of AP activity, cell colonies fixed in 4% paraformaldehyde (20 min, room temperature) were stained with an AP solution (Sigma-Aldrich; Merck KGaA) for 30 min at room temperature. Following a 30-min incubation, the colonies were washed three times with PBS, and the observations of the colonies were conducted.

Bat induced pluripotent stem cells (BatiPSCs) were dissociated into single cells using 0.1% trypsin/1 mM EDTA solution and resuspended in differentiation medium composed of DMEM supplemented with 10% FBS and 1% non-essential amino acids. The cells were plated onto non-adherent bacterial-grade petri dishes at a density of 2 × 10^6^ cells per dish in 10 ml differentiation medium. To achieve uniform distribution of cells, the Petri dish underwent gentle agitation and was subsequently transferred to a humidified incubator set at 37°C with 5% CO_2_. Following a 24-h incubation period, the medium was substituted with a fresh differentiation medium to eliminate non-adherent cells. During the initial 4 days, embryoid bodies were kept in suspension culture using pluripotent stem cell culture medium without Leukaemia inhibitory factor (LIF). The culture medium was refreshed every two days and the cells were maintained in culture for a total duration of 7 days.

### 2.6. Teratoma formation

The cells were grown to confluence and dissociated. A total of 4 × 10^6^ cells diluted in 1/4 Matrigel were injected subcutaneously in the right dorsal flank of 6–8 week-old male BALB/c nude mice (*n* = 6) (obtained from Japan SLC, Inc.). The subcutaneous tumour growth progression was observed every two days. The experiment was conducted in accordance with the indicator that if problems such as eating or movement limitations due to the tumour occurred, the experiment would have been terminated immediately. Following tumour formation, the animals were euthanized in a CO_2_ atmosphere chamber and euthanized by cervical dislocation. The resultant teratoma was histologically assessed using haematoxylin and eosin staining to examine the endoderm derivatives [22]; moreover, Masson’s trichrome stain was used to examine the muscle fibres or mesoderm derivatives [23,24] and a periodic acid-Schiff stain to examine the ectoderm epithelium [25].

### 2.7. Quantitative real-time PCR

Invitrogen TRIzol® reagent was used for RNA extraction. On the day of RNA extraction, BEF, human iPSCs (Cat #ACS-1011, ATCC, Manassas, VA, USA), and BatiPSCs were harvested and treated with 200 ml TRIzol® reagent. RNA extraction was performed following the instructions provided by the manufacturers. Two hundred ng of isolated total RNAs were converted to cDNA in 20-µl reaction volumes by using a Moloney murine leukaemia virus cDNA synthesis kit from Enzynomics, according to the manufacturer’s instructions. Reverse transcription-quantitative PCR (RT-qPCR) was performed by diluting the cDNA samples five times and 3 ml of the diluted cDNA (30 ng or 6 ng) was transferred to each well of a 96-well reaction plate (Applied Biosystems; Thermo Fisher Scientific, Inc.). TOPreal qPCR 2 PreMIX (SYBR green with low ROX, Enzynomics) served as a fluorescent signal to detect the target cDNA amounts. The thermal cycling protocol involved an initial step at 95°C for 10 minutes, followed by 40 cycles comprising 10 seconds at 95°C, 20 seconds at 60°C, and 40 seconds at 72°C. The relative mRNA expression levels were determined by the ΔΔCt method [26], with GAPDH used as endogenous control. Primers’ sequences and accession numbers are provided in Table 1 [1,15]. The reactions were run on a StepOnePlus real-time PCR system (Applied Biosystems; Thermo Fisher Scientific, Inc.).

**Table 1.** Primers used for relative quantitative PCR, according to the reference of Mo et al. [15] and Déjosez et al. [1].

Gene	F	R	Product size	Accession
Oct4/POU5F1	GGTACACCCAGGCCGATGT	GATGGTCGTTTGGCTGAACA	71	XM_059702114.1
SOX2	CTGCGAGCGCTGCACAT	TCATGAGCGTCTTGGTTTTCC	73	XM_036352470.1
MYC	ACGTCAGCTTCGCCAACAG	GTTCTCTTCCTCGTCGCAGAA	80	XM_036337663.1
KLF4	CGAACCCACACAGGTGAGAAA	CTGAGCGGGCAAACTTCCA	70	XM_036330103.1
GAPDH	TGGTGAAGGTCGGAGTGAAC	GAAGGGGTCATTGATGGCGA	104	XM_036322999.1

### 2.8. Generation of bat-mice chimera in nude mouse by using blastocyst complementation

The injection of bat-iPS cells into mouse blastocysts was performed as previously described [27]. Eight-cell embryos were flushed from the oviducts of plugged females at 2.5 dpc in medium 2 (M2; MilliporeSigma). The collected embryos were cultured on blastocyst stage at 37°C with 5% CO_2_ in KSOM medium. Embryo injections were performed on blastocysts stage. 12–15 Bat-iPS cells were injected into blastocysts. Following injection, the blastocysts were incubated in potassium simplex optimized medium with amino acids (KSOM-AA; MilliporeSigma) at 37°C until transferred to the uterine of pseudo-pregnant Swiss albino female mice by standard methods.

### 2.9. Statistical analysis

The data are shown as mean ± SEM (standard error of the mean). The RT-qPCR data combined triplicates from one independent experiment. The differentiation experiments were replicated in two independent experiments with triplicate samples. One way ANOVA test was used to compare the three groups and Tukey’s post-hoc test was used to determine the difference among the groups. The asterisks were used to indicate the statistical significance between the groups (*p < 0.05, **p < 0.01, ***p < 0.001)

## 3. Results

### 3.1. Establishment of bat fetal fibroblast cell lines and generation of BatiPSCs

**Figure 1.**
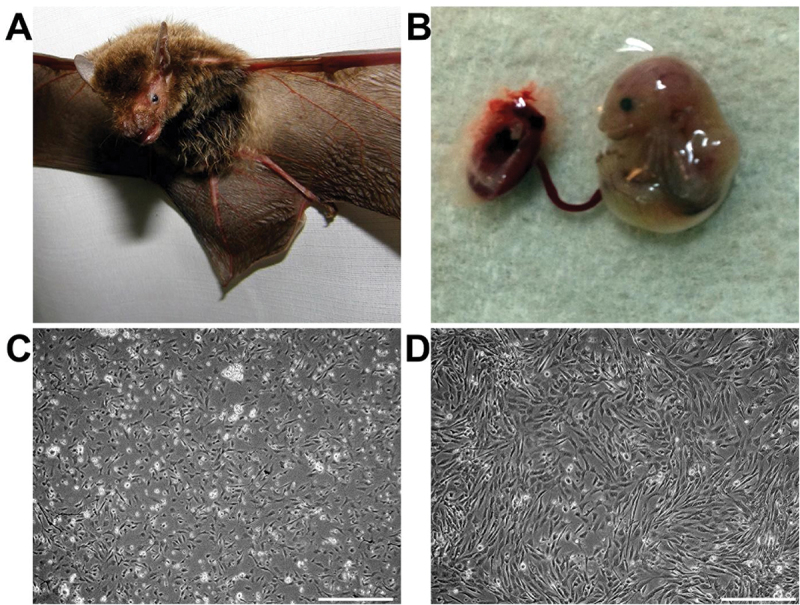
Establishment of bat foetal fibroblast cell line. (A) Pregnant bat was used to obtain the embryos (B), and embryonic fibroblasts were obtained, (C) in growing stages, and (D) at confluency. Scale bar = 250 µm.

**Figure 2.**
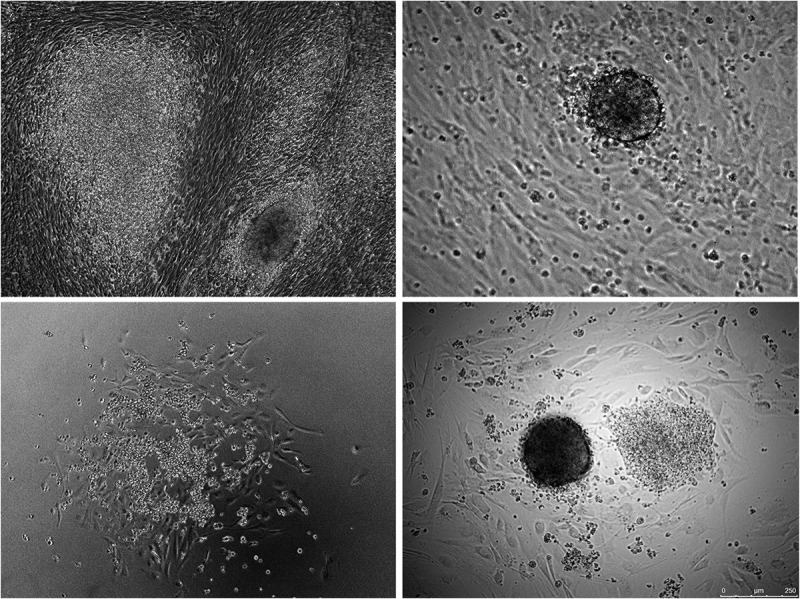
Generation of BatiPSC. From the 9th day onward, different types of colonies derived from bat-induced pluripotent stem cells (BatiPSC) started appearing. Notably, we successfully acquired colonies exhibiting morphological features reminiscent of primate iPSCs in plate shape and rodent iPSCs in dome shape. Scale bar = 250 µm.

To generate BatiPSCs, BEFs (Figure 1(A-D)) underwent transfection using integration-free CytoTune®-iPS 2.0 Sendai Reprogramming Kit. Typically, the CytoTune Kit enables colonies to be formed approximately on the 12th day. However, in the case of bat cells, iPSC-like colonies were observed approximately 3 days earlier than the standard timeline (depicted in Figure 2). Colonies exhibiting a compact, glossy appearance with distinct edges and a 3D structure were manually selected and expanded following trypsinization.

In order to identify suitable culture conditions for BatiPSCs, two commonly used culture media for human and mouse stem cells, TeSR™-E8™ and Stempan E14 GMEM, were employed. On the fourth day of culture, it was observed that cells in the Stempan E14 GMEM medium were well-maintained, while cells in the TeSR™-E8™ medium exhibited signs of degeneration. The most significant difference between TeSR™-E8™ and Stempan E14 GMEM media lies in the presence or absence of LIF. Therefore, LIF is considered to be a crucial factor for the maintenance of BatiPSCs.

### 3.2. Characterization of BatiPSCs

**Figure 3.**
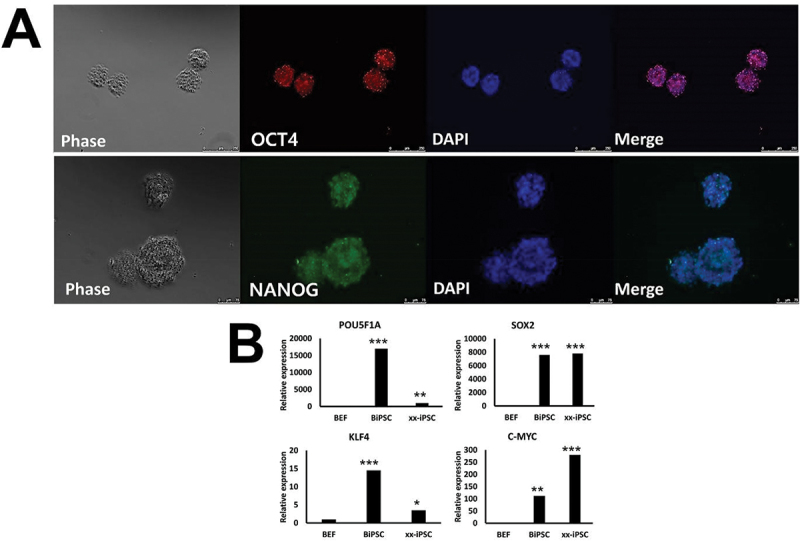
Immunofluorescence and qPCR characterization of BatiPSC. To identify the cell type most closely resembling iPSCs, we conducted OCT4 (scale bar = 250 µm) and nanog (scale bar = 75 µm) immunofluorescence staining (A) and RT-qPCR (B). The findings demonstrated strong expression of pluripotent stem cell markers, particularly in colonies with a dome-shaped structure. Asterisks were used to indicate statistical significance between the groups (*p < 0.05, **p < 0.01, ***p < 0.001).

**Figure 4.**
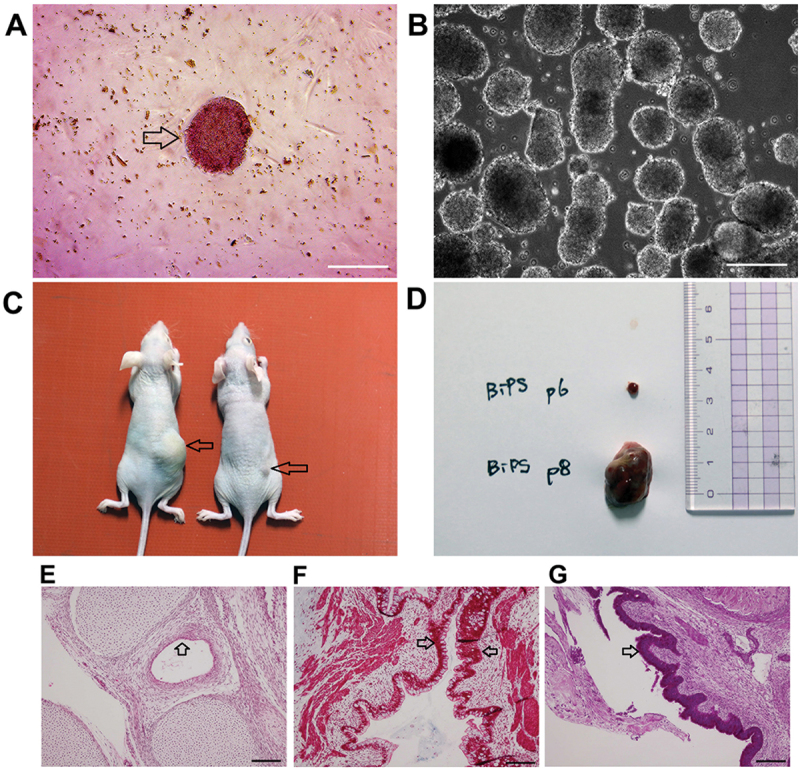
Characterization of bat iPSC through AP reaction (A), embryoid bodies (B) and teratoma formation (C-D). Scale bar = 75 µm. Histological sections of the resultant teratoma revealed (E) endoderm (arrow head) (stained with H&E stain; haematoxylin-stained nuclei in a purplish-blue hue, while eosin imparted a pink colouration to the extracellular matrix and cytoplasm), (F) mesoderm (stained red with Masson’s trichrome stain, arrow head), and (G) ectoderm (stained magenta with PAS stain, arrow head). Scale bar = 100 µm.

BatiPSCs expressed the pluripotency factors Oct4 (POU5F1A), Sox2 and Nanog as determined by immunofluorescence staining (Figure 3(A)). Using qPCR, the expression levels of pluripotency markers were compared in BEFs and human iPSCs. A marked increase was noted in the expression levels of Oct4 in BatiPSCs, while the expression levels of c-Myc were significantly lower in BatiPSCs (Figure 3(B)). Furthermore, BatiPSCs indicated positive staining of AP, which is a marker of pluripotency (Figure 4(A)); the data indicated the ability of BatiPSCs to form embryoid bodies following culture in low-attachment dishes (Figure 4(B)). It is interesting to note that BatiPSCs were able to induce teratoma following subcutaneous injection in non-immune nude mice. About 2 cm of teratoma growth were formed in five mice, except for one mouse which showed a small sized teratoma (Figure 4(C,D)). Histological sections of the resultant teratoma revealed differentiation of the injected cells into the three germ layers. H&E-stained sections indicated gut-like epithelial cells where haematoxylin-stained nuclei in a purplish-blue hue, while eosin imparted a pink colouration to the extracellular matrix and cytoplasm, representing endoderm derivatives (Figure 4(E)). Muscle fibres stained red with Masson’s trichrome stain, representing mesoderm derivatives (Figure 4(F)). Furthermore, PAS stain showed positively magenta-coloured secretory epithelium, representing ectoderm derivatives (Figure 4(G)).

### 3.3. Generation of green fluorescent protein (GFP) expressing BatiPSCs and their use for chimeric mouse production

**Figure 5.**
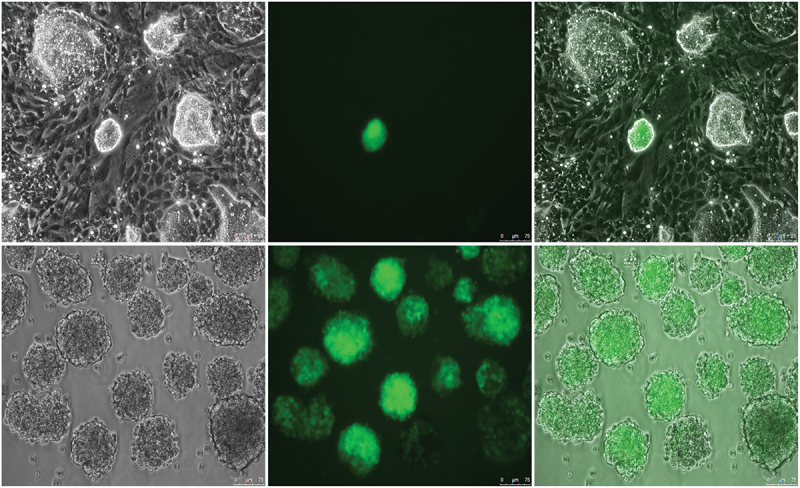
Generation of GFP bat iPSC. GFP was transfected to BatiPSCs colonies and positive colonies were used for further passaging and expanding the culture. Scale bar = 75 µm.

**Figure 6.**
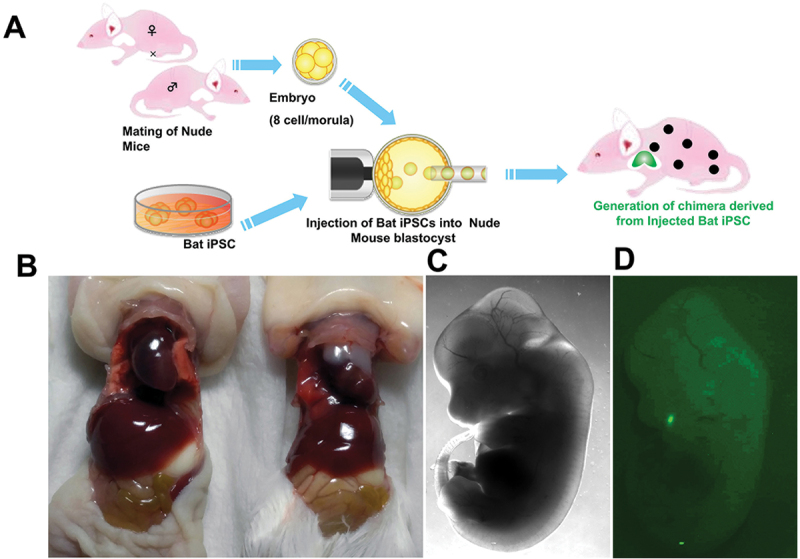
Generation of bat-mice chimera in nude mice through using blastocyst complementation. (A) method of blastocyst complementation through injecting bat iPSCs into mice blastocysts. (B-D) Chimeric embryos were not observed however, we observed a slight GFP signal at the neck region.

BatiPSCs GFP-expressing colonies were selected and further expanded in culture (Figure 5). These GFP-expressing cells were injected into the blastocele of wild nude mouse blastocysts. The resultant blastocysts were transferred to surrogate mice and the foetuses were examined with regard to the expression of GFP. The results indicated that GFP signals were slightly detected in the neck region of the mouse foetuses (Figure 6; supplementary STable 1).

## 4. Discussion

The conservation of Asian bats, particularly those surviving in Korea, is crucial for maintaining broader ecosystem functions and resilience. This conservation effort ensures the sustainability of natural habitats and the services they provide to humans and other wildlife [28,29]. In our current investigation, we aimed to generate Bat-induced Pluripotent Stem Cells (BatiPSCs) as a tool for conserving two Korean native bat species, *Hypsugo alaschanicus* and *Pipistrellus abramus*, and as an alternative model for live animal research.

As indicated in Table 2, there was a reasonable similarity between the amino acid composition of pluripotency factors in bats and humans compared to their mouse counterparts. To establish BatiPSC cell lines, we utilized the integration-free CytoTune®-iPS 2.0 Sendai Reprogramming Kit in conjunction with a suitable cell culture system. This Kit offers several advantages over other methods of iPSC production, primarily due to its non-integrative nature and high efficiency. Unlike methods that integrate into the host genome, the Sendai virus-based system maintains the genomic integrity of reprogrammed cells, reducing the risk of insertional mutagenesis and subsequent genetic instability or tumorigenesis [30]. The transient expression of viral RNA, which is diluted out as cells divide, eliminates concerns about long-term expression of reprogramming factors [31]. Additionally, the CytoTune®-iPS 2.0 Kit is known for its high reprogramming efficiency, often surpassing other non-integrative methods such as episomal vectors or mRNA-based reprogramming, resulting in faster generation of iPSCs and a higher yield of reprogrammed colonies [32]. Several recent reports have shown the merits of using the CytoTune®-iPS 2.0 Kit for generating iPSCs from human and animal species due to its improved efficiency, safety, and ease of use [33–36]. These advantages are crucial for both research and potential clinical applications in regenerative medicine.

**Table 2.** Amino acid identity comparison between bat, human, and mouse reprogramming factors.

Protein	Human	Mouse
Oct4	87%	82%
Sox2	96%	94%
Klf4	94%	91%
Myc	78%	79%

The current results showed the essential roles of LIF in inducing and maintaining pluripotency during the generation of BatiPSCs, ensuring the stability and robustness of reprogrammed cells [37]. LIF activates the JAK-STAT3 signalling pathway, which in turn upregulates key pluripotency-associated genes such as Oct4, Sox2, and Nanog, thereby maintaining the cells in an undifferentiated state [38,39].

Starting from the 9^th^ day of culture, various forms of BatiPSC colonies began to emerge. Among these, colonies with morphological characteristics resembling the plate shape of primate iPSCs and the dome shape typical of rodent iPSCs were identified. To determine which cell type closely resembled iPSCs, AP staining and immunofluorescence staining for pluripotency markers were performed. The results revealed a robust expression of pluripotent stem cell markers, particularly in the dome-shaped colonies. The BatiPSCs demonstrated specific characteristics akin to embryonic stem cells (ESCs), such as typical iPSC morphology, positive AP staining, expression of pluripotency markers, and the ability to form embryoid bodies [6,7].

Xenotransplantation experiments were conducted involving bats and mice; however, clear chimeric results were not obtained. The rationale for utilizing nude mice was based on their congenital deficiency in thymic tissue, making them a representative model of organ deficiency [40]. The hypothesis was that the introduction of BatiPSCs into nude mouse embryos may lead to the regeneration of thymic tissue derived from bat-induced pluripotent stem cells during embryonic development. However, chimeric formation was not observed in either wild-type mice or nude mice. Previous studies have suggested that specific differentiated stem cells are necessary to create chimeras between different species [41]. It is anticipated that intermediate-stage differentiated stem cells will be required to establish chimeras between bats and mice.

The emergence of BatiPSC colonies after approximately 9 days of cultivation signifies the efficiency of the reprogramming process. Immunofluorescence staining demonstrated the expression of key pluripotency factors, Oct4 (POU5F1A) and Nanog, affirming their stem cell identity. The RT-qPCR results demonstrated a substantial increase in Oct4 expression, distinguishing BatiPSCs from the original BEFs and human iPSCs, indicating that the BiPSC lines consistently retained an embryonic stem cell state [42]. The expression levels of c-Myc were significantly reduced in BatiPSCs, indicating the successful enhancement of the pluripotency of iPSC [43]; this evidence suggests a unique molecular profile that warrants further investigation.

Positive AP staining, a recognized pluripotency marker, further confirmed the undifferentiated state of BatiPSCs [44,45]. The successful formation of embryoid bodies in low-attachment dishes demonstrated their ability to undergo spontaneous differentiation, a characteristic feature of pluripotent stem cells [46]. Notably, the induction of teratoma in non-immune nude mice following subcutaneous injection underlines the tumorigenic potential of BatiPSCs, indicating their capability to differentiate into tissues representative of all three germ layers [47,48]. This was also confirmed also by the detection of GFP signals in the neck region of the chimeric mouse foetuses, underlining the potential of BatiPSCs to contribute to the development of specific tissues and organs *in vivo* [41,49].

Contrary to previously reported bat iPSC lines, which used different species: *Hypsugo alaschanicus*, *Pipistrellus abramus*, *Rhinolophus ferrumequinum*, and *Myotis myotis* as reported by Déjosez et al. [1], and *Myotis Lucifugus* as reported by Qin et al. [2]. We used different integration-free system for expressing the pluripotency factors [1,2,15], the method presented in the current study confirmed the ability of BatiPSCs to form differentiating teratoma and integrate into the blastocysts. Nevertheless, additional molecular characterization of both PBFF and the resultant BatiPSCs is necessary.

In conclusion, the establishment and characterization of BatiPSCs, coupled with their successful integration into chimeric mice, underscore their potential as valuable tools for the preservation of endangered bat species. The distinctive molecular profile of BatiPSCs and their demonstrated pluripotency render them promising for conservation efforts. Further studies are required to obtain bat naïve iPSCs and exploration of other bat species for refinement of interspecies chimera production using BatiPSCs.

## Supplementary Material

Sup table.1.docx

## Data Availability

The data supporting the findings of this study is available from the corresponding author upon a reasonable request.

## References

[1] Déjosez M, Marin A, Hughes GM, et al. Bat pluripotent stem cells reveal unusual entanglement between host and viruses. Cell. 2023;186(5):957–974.e928. doi: 10.1016/j.cell.2023.01.011 36812912 PMC10085545

[2] Qin Y, Li C, Gao X, et al. Derivation of transgene-free bat induced pluripotent stem cells amenable to chimera formation in mice, pigs, and chicks. Cell Discov. 2023;9(1). doi: 10.1038/s41421-023-00587-3 PMC10480176 37669932

[3] Wu Y, Wang C, Fan X, et al. The impact of induced pluripotent stem cells in animal conservation. Vet Res Commun. 2024;48:649–663. doi: 10.1007/s11259-024-10294-3 38228922

[4] Kasso M, Balakrishnan M. Ecological and economic importance of bats (order Chiroptera). ISRN Biodivers. 2013;2013:1–9. doi: 10.1155/2013/187415

[5] Ye L, Swingen C, Zhang J. Induced pluripotent stem cells and their potential for basic and clinical sciences. Curr Cardiol Rev. 2013;9(1):63–72. doi: 10.2174/157340313805076278 22935022 PMC3584308

[6] Takahashi K, Yamanaka S. Induction of pluripotent stem cells from mouse embryonic and adult fibroblast cultures by defined factors. Cell. 2006;126(4):663–676. doi: 10.1016/j.cell.2006.07.024 16904174

[7] Takahashi K, Tanabe K, Ohnuki M, et al. Induction of pluripotent stem cells from adult human fibroblasts by defined factors. Cell. 2007;131(5):861–872. doi: 10.1016/j.cell.2007.11.019 18035408

[8] Chehelgerdi M, Behdarvand Dehkordi F, Chehelgerdi M, et al. Exploring the promising potential of induced pluripotent stem cells in cancer research and therapy. Mol Cancer. 2023;22(1). doi: 10.1186/s12943-023-01873-0 PMC10683363 38017433

[9] Karami Z, Moradi S, Eidi A, et al. Induced pluripotent stem cells: generation methods and a new perspective in COVID-19 research. Front Cell Dev Biol. 2023;10. doi: 10.3389/fcell.2022.1050856 PMC9887183 36733338

[10] Stadtfeld M, Nagaya M, Utikal J, et al. Induced pluripotent stem cells generated without viral integration. Science. 2008;322(5903):945–949. doi: 10.1126/science.1162494 18818365 PMC3987909

[11] Hao YJ, Wang YL, Wang MY, et al. The origins of COVID‐19 pandemic: a brief overview. Transbound Emerg Dis. 2022;69(6):3181–3197. doi: 10.1111/tbed.14732 36218169 PMC9874793

[12] Park G, Rim YA, Sohn Y, et al. Replacing animal testing with stem cell-Organoids: advantages and limitations. STEM Cell Rev And Rep. 2024;2024: doi: 10.1007/s12015-024-10723-5 PMC11319430 38639829

[13] Pessôa LVDF, Bressan FF, Freude KK. Induced pluripotent stem cells throughout the animal kingdom: availability and applications. World J Stem Cells. 2019;11(8):491–505. doi: 10.4252/wjsc.v11.i8.491 31523369 PMC6716087

[14] Kim T-W, Che J-H, Yun J-W. Use of stem cells as alternative methods to animal experimentation in predictive toxicology. Regul Toxicol Pharmacol. 2019;105:15–29. doi: 10.1016/j.yrtph.2019.03.016 30930176

[15] Mo X, Li N, Wu S. Generation and characterization of bat-induced pluripotent stem cells. Theriogenology. 2014;82(2):283–293. doi: 10.1016/j.theriogenology.2014.04.001 24853281 PMC7103130

[16] Haarsma A-J. Manual for assessment of reproductive status, age and health in European vespertilionid bats. Hillegom, Holland: Electronic publication Version 1; 2008.

[17] Ellis MV. Development of a compact system for field euthanasia of small mammals. J Mammal. 2017;98(4):1211–1214. doi: 10.1093/jmammal/gyx072

[18] Reilly JS. Euthanasia of animals used for scientific purposes. 2nd ed. Adelaide: ANZCCART, Adelaide University; 2001.

[19] Group MRW. Humane euthanasia of bats for public health rabies testing. Lansing (MI): Michigan Rabies Working Group; 2014. p. 2014.

[20] Altringham JD. Reproduction and development. Bats. 2011;10(1093/acprof:osobl/9780199207114.003.0005pp):113–135.

[21] Badwaik NK, Rasweiler JJ. (6) Pregnancy. In: Reproductive biology of bats. Academic Press; 2000. p. 221–293. doi: 10.1016/b978-012195670-7/50007-2

[22] Nelakanti RV, Kooreman NG, Wu JC. Teratoma formation: a tool for monitoring pluripotency in stem cell research. Curr Protoc Stem Cell Biol. 2015;32(1). doi: 10.1002/9780470151808.sc04a08s32 PMC4402211 25640819

[23] Zhai M, Zhu Y, Yang M, et al. Human mesenchymal stem cell derived exosomes enhance cell‐Free bone regeneration by altering their miRNAs profiles. Adv Sci. 2020;7(19). doi: 10.1002/advs.202001334 PMC7539212 33042751

[24] Lee M-O, Moon SH, Jeong H-C, et al. Inhibition of pluripotent stem cell-derived teratoma formation by small molecules. In: *Proceedings of the National Academy of Sciences*; 2013, 110, doi:10.1073/pnas.1303669110 PMC3761568 23918355

[25] Kim JS, Hong YJ, Choi HW, et al. Generation of in vivo neural stem cells using partially reprogrammed cells defective in in vitro differentiation potential. Oncotarget. 2017;8(10):16456–16462. doi: 10.18632/oncotarget.14861 28147316 PMC5369976

[26] Livak KJ, Schmittgen TD. Analysis of relative gene expression data using real-time quantitative PCR and the 2−ΔΔCT method. Methods. 2001;25(4):402–408. doi: 10.1006/meth.2001.1262 11846609

[27] Nagy A, Gertsenstein M, Vintersten K; Behringer R, editors, editors. Manipulating the mouse embryo; a laboratory manual. *3* ed. (NY): Cold Spring Harbor Laboratory Press; 2003.

[28] Jo Y-S, Baccus JT, Koprowski JL. Mammals of Korea: a review of their taxonomy, distribution and conservation status. Zootaxa. 2018;4522(1). doi: 10.11646/zootaxa.4522.1.1 30486139

[29] Yoon KB, Lim SJ, Park YC. Analysis on habitat characteristics of the Korean bats (Chiroptera) using geographic information system (GIS). J For And Environ Sci. 2016;32(4):377–383. doi: 10.7747/jfes.2016.32.4.377

[30] Lieu PT, Fontes A, Vemuri MC. Generation of induced pluripotent stem cells with CytoTune, a non-integrating Sendai virus. Methods Mol Biol. 2013;997:45–56. doi: 10.1007/978-1-62703-348-0_5 23546747

[31] Kunitomi A, Hirohata R, Arreola V, et al. Improved Sendai viral system for reprogramming to naive pluripotency. Cell Rep Methods. 2022;2(11):100317. doi: 10.1016/j.crmeth.2022.100317 36447645 PMC9701587

[32] Beers J, Linask KL, Chen JA, et al. A cost-effective and efficient reprogramming platform for large-scale production of integration-free human induced pluripotent stem cells in chemically defined culture. Sci Rep. 2015;5(1). doi: 10.1038/srep11319 PMC4464084 26066579

[33] Haase A, Göhring G, Martin U. Generation of non-transgenic iPS cells from human cord blood CD34 + cells under animal component-free conditions. Stem Cell Res. 2017;21:71–73. doi: 10.1016/j.scr.2017.03.022 28677540

[34] Ura H, Togi S, Hatanaka H, et al. Establishment of a human induced pluripotent stem cell line, KMUGMCi010-A, from a patient with X-linked ohdo syndrome bearing missense mutation in the MED12 gene. Stem Cell Res. 2024;77:103388. doi: 10.1016/j.scr.2024.103388 38492468

[35] Shi Z, Liu H, Feng F, et al. Generation of an induced pluripotent stem cell line GWCMCi006-A from a patient with autosomal dominant neurodevelopmental disorder with or without hyperkinetic movements and seizures harboring GRIN1 c.389A > G mutation. Stem Cell Res. 2024;76. doi: 10.1016/j.scr.2024.103371 38452705

[36] Ishikawa K-I, Shiga T, Yoshino H, et al. Generation of three clones (JUCGRMi002-A, B, C) of induced pluripotent stem cells from a Parkinson’s disease patient with SNCA duplication. Stem Cell Res. 2024;74:103296. doi: 10.1016/j.scr.2023.103296 38154385

[37] Cooney AJ, Hirai H, Firpo M, et al. Establishment of LIF-Dependent human iPS cells closely related to basic FGF-Dependent authentic iPS cells. PLOS ONE. 2012;7(6):e39022. doi: 10.1371/journal.pone.0039022 22720020 PMC3374774

[38] Telugu BPVL, Ezashi T, Sinha S, et al. Leukemia inhibitory factor (LIF)-dependent, pluripotent stem cells established from inner cell Mass of porcine embryos. J Biol Chem. 2011;286(33):28948–28953. doi: 10.1074/jbc.M111.229468 21705331 PMC3190702

[39] Graf U, Casanova EA, Cinelli P. The role of the leukemia inhibitory factor (LIF) — pathway in derivation and maintenance of murine pluripotent stem cells. Genes (Basel). 2011;2(1):280–297. doi: 10.3390/genes2010280 24710148 PMC3924847

[40] Mecklenburg L, Tychsen B, Paus R. Learning from nudity: lessons from the nude phenotype. Exp Dermatol. 2005;14(11):797–810. doi: 10.1111/j.1600-0625.2005.00362.x 16232301

[41] Wu J, Platero-Luengo A, Sakurai M, et al. Interspecies chimerism with mammalian pluripotent stem cells. Cell. 2017;168(3):473–486.e415. doi: 10.1016/j.cell.2016.12.036 28129541 PMC5679265

[42] Hanna JH, Saha K, Jaenisch R. Pluripotency and cellular reprogramming: facts, hypotheses, unresolved issues. Cell. 2010;143(4):508–525. doi: 10.1016/j.cell.2010.10.008 21074044 PMC3032267

[43] Habib O, Habib G, Choi HW, et al. An improved method for the derivation of high quality iPSCs in the absence of c-myc. Exp Cell Res. 2013;319(20):3190–3200. doi: 10.1016/j.yexcr.2013.09.014 24095950

[44] Štefková K, Procházková J, Pacherník J. Alkaline phosphatase in stem cells. Stem Cells Int. 2015;2015:1–11. doi: 10.1155/2015/628368 PMC4342173 25767512

[45] O’Connor MD, Kardel MD, Iosfina I, et al. Alkaline phosphatase-positive colony formation is a sensitive, specific, and quantitative indicator of undifferentiated human embryonic stem cells. Stem Cells. 2008;26(5):1109–1116. doi: 10.1634/stemcells.2007-0801 18276800

[46] Guo N-N, Liu L-P, Zheng Y-W, et al. Inducing human induced pluripotent stem cell differentiation through embryoid bodies: a practical and stable approach. World J Stem Cells. 2020;12:25–34. doi: 10.4252/wjsc.v12.i1.25 32110273 PMC7031760

[47] Gutierrez-Aranda I, Ramos-Mejia V, Bueno C, et al. Human induced pluripotent stem cells develop teratoma more efficiently and faster than human embryonic stem cells regardless the site of injection. Stem Cells. 2010;28(9):1568–1570. doi: 10.1002/stem.471 20641038 PMC2996086

[48] Hong SG, Winkler T, Wu C, et al. Path to the clinic: assessment of iPSC-based cell therapies in vivo in a nonhuman Primate Model. Assess Of iPSC-Based Cell Therapies In Vivo In a Nonhum Primate Model. Cell Rep. 2014;7(4):1298–1309. doi: 10.1016/j.celrep.2014.04.019 24835994 PMC4058889

[49] West FD, Terlouw SL, Kwon DJ, et al. Porcine induced pluripotent stem cells produce chimeric offspring. Stem Cells Dev. 2010;19(8):1211–1220. doi: 10.1089/scd.2009.0458 20380514

