# Supplementary material for: Generation of induced pluripotent stem cells from the Asian bats
Source: Int J Vet Sci Med. 2024 Aug 12;12(1):81–90. doi: 10.1080/23144599.2024.2384835 (PMC11321101; doi:10.1080/23144599.2024.2384835)
Supplement: Sup table.1.docx [file TVSM_A_2384835_SM8699.docx]

| Embryo Donor Strain | No. Transferred blastocyst | No. Neonate (%) | No. Chimera (%) | No. Generated thymus (%) | |
| --- | --- | --- | --- | --- | --- |
| ICR | 184 | 109 (59) | 0 (0) | | - |
| Nude/Balb/C | 133 | 65 (48) | 0 (0) | | 0 (0) |

Supplementary Table1.

**Result of chimeric mice and generated thymus.**
